# Twins Early Development Study (TEDS): A genetically sensitive investigation of mental health outcomes in the mid‐twenties

**DOI:** 10.1002/jcv2.12154

**Published:** 2023-03-30

**Authors:** Celestine Lockhart, Joanna Bright, Yasmin Ahmadzadeh, Gerome Breen, Shannon Bristow, Andy Boyd, Johnny Downs, Matthew Hotopf, Elisavet Palaiologou, Kaili Rimfeld, Jessye Maxwell, Margherita Malanchini, Tom A. McAdams, Andrew McMillan, Robert Plomin, Thalia C. Eley

**Affiliations:** ^1^ Social, Genetic and Developmental Psychiatry Centre Institute of Psychiatry, Psychology and Neuroscience King's College London Camberwell London UK; ^2^ UK National Institute for Health Research (NIHR) Biomedical Research Centre South London and Maudsley Hospital London UK; ^3^ Population Health Sciences Institute Bristol Medical School University of Bristol Bristol UK; ^4^ Department of Child and Adolescent Psychiatry Institute of Psychiatry, Psychology & Neuroscience King's College London London UK; ^5^ South London and Maudsley NHS Foundation Trust London UK; ^6^ Department of Psychological Medicine Institute of Psychiatry Psychology and Neuroscience King's College London London UK; ^7^ Department of Psychology Royal Holloway University of London Egham Surrey UK; ^8^ Queen Mary University of London London UK; ^9^ Promenta Research Centre University of Oslo Oslo UK

**Keywords:** behavioural genetics, longitudinal studies, mental health, TEDS, twins

## Abstract

The Twins Early Development Study (TEDS) is a longitudinal study following a cohort of twins born 1994–1996 in England and Wales. Of the 13,759 families who originally consented to take part, over 10,000 families remain enrolled in the study. The current focus of TEDS is on mental health in the mid‐twenties. Making use of over 25 years of genetically sensitive data, TEDS is uniquely placed to explore the longitudinal genetic and environmental influences on common mental health disorders in early adulthood. This paper outlines recent data collection efforts supporting this work, including a cohort‐wide mental health assessment at age 26 and a multi‐phase Covid‐19 study. It will also provide an update on data linkage efforts and the Children of TEDS (CoTEDS) project.

**Key points**

- The Twins Early Development Study (TEDS) is a longitudinal cohort study of twins born in England and Wales between 1994 and 1996.
- TEDS has investigated the genetic and environmental influences on cognition, behaviour, and emotion for over 25 years.
- Now that the twins have reached their mid‐20s, a peak age for the onset of common mental health disorders, the focus of TEDS is on understanding the development and onset of mental health symptoms.
- TEDS has undertaken various data collections in this area, including a cohort wide mental health assessment at age 26, a COVID‐19 study, data linkage, and with the development of the Children of TEDS (CoTEDS) project.
- TEDS data is free to access, and the team welcomes collaborations with external researchers.

## INTRODUCTION

The Twins Early Development Study (TEDS) is a longitudinal study which has followed a cohort of twins born between 1994 and 1996 in England and Wales for over 25 years. With data collected between ages 1 and 26 years, TEDS has investigated the genetic and environmental influences on individual differences in cognition, mental health, and behaviour. Now that the participants have reached adulthood, the study is uniquely placed to examine how these developmental experiences influence a range of adult outcomes. In addition to information collected from TEDS twins and their parents, children of the TEDS twins are now being recruited into the Children of the Twins Early Development Study (CoTEDS). This paper follows previous sample descriptions (Ahmadzadeh et al., 2019; Haworth et al., 2013; Oliver & Plomin, 2007; Rimfeld et al., 2019; Trouton et al., 2002) by providing an update on current sample characteristics and discussing recent and planned data collection.

### Initial recruitment, data collection and sample characteristics

Families in England and Wales with twins born between January 1994 and December 1996 were identified using electronic birth records and invited to join TEDS through the Office of National Statistics (ONS). According to data from the ONS, there would have been approximately 30,350 multiple births between 1994 and 1996 (Birth Characteristics, 2023; Vital Statistics in the UK, 2021). Of the families contacted, 16,810 parents expressed interest in registering their twins to be part of the study, with 13,759 consenting to take part during the first wave when twins were 18 months old. This initial data collection assessed perinatal characteristics, family demographics and twin zygosity. Parent reported zygosity using questionnaires has been found to be 95% accurate (Price et al., 2000). However, DNA testing was offered in cases where zygosity was unclear.

Since first contact, data have been collected at 2, 3, 4, 7, 8, 9, 10, 12, 14, 16, 18, 21, and most recently, 26 years. However, budgetary restraints meant the complete sample was not invited to take part in every wave. At ages where the budget was more constrained, the older school cohorts, which contained the majority of TEDS participants, were prioritised. Core waves of assessment, in which the full contactable sample are invited, took place at ages 4, 7, 8, 12, 14, 16, 18, 21 and 26 years. Overall changes in sample size can be seen in Supplementary Figure 1. Data have been collected via paper questionnaires, phone calls, in‐person visits, with mobile apps and online testing platforms. Parents, twins, and teachers have all provided data at various time points, creating a rich multi‐informant database. Data linkage efforts, including education records and environmental measures linked using the unique postcodes associated with home addresses, have further extended the TEDS dataset. Twins' parents provided informed consent at each wave of study participation until age 16, after which twins were asked directly. The TEDS team keeps in touch with participants using our social media and annual newsletter and has also undertaken participant focus groups to inform our future research plans.

To encourage participation, depending on the scale of data collection, small rewards and prize draws are offered. For core waves both rewards and regular prize draws are offered. Core waves require extended data collection periods and team resources, including regular reminders, phone calls and postal paper questionnaires. Other waves of assessment typically receive fewer resources and rely solely on prize draws. Such assessments usually have reduced timeframes and result in lower data returns.

As with any longitudinal cohort study, the TEDS sample has experienced attrition since first contact. However, over 10,000 families remain involved in the study, with more than 6000 families providing data at both ages 21 and 26 (seen in Table 1). Parent‐reported family characteristics have remained largely stable since first contact. However, the proportion of the sample with parents with a qualification level of A‐levels or higher and in employment at first contact has increased slightly.

**TABLE 1:** Changes in the socio‐demographic characteristics of the TEDS sample since first contact.

	Returned data (*N* families)	Age of twins (mean)	Full twin pairs (*N*)	Female (%)	MZ (%)	White ethnic groups (%)	Mothers with A‐levels or higher (%)	Fathers with A‐levels or higher (%)	Mother employed (%)	Father employed (%)
First contact	13,101	–	12,939	50.3	33.6	92.0	35.7	45.1	43.2	91.8
Age 21	6670	22.3	4369	61.4	36.4	93.5	44.7	52.0	47.4	93.9
Age 26	6296	26.4	4558	65.4	36.8	93.9	44.4	51.9	46.8	93.7

*Note*: Total data returns at age 26 include any family who contributed data at any data collection since age 21. Family characteristics at each age are compared to those collected at first contact. Twin and family socio‐demographic characteristics reported in this table are based on parent reports at first contact.

At first contact, the proportion of the TEDS sample belonging to ethnic minority groups reflected the UK national population in the mid‐1990s. Parents reported 92.0% of twins to be in ‘White’ ethnic groups, compared to 93.0% of the UK population (Walker et al., n.d.). Based on parent‐reports, attrition has been higher among ethnic minority participants, with 93.9% of twins at age 26 belonging to a ‘White’ ethnic group (‘White British’, ‘White Irish’, or ‘Other White’). Twin self‐reported ethnicity at age 26 corresponds with parent reports, with 94.3% of twins identifying as White. Self‐reported ethnicity at age 26 is shown in Table 2.

**TABLE 2:** Self‐reported ethnicity at age 26.

	*N*	Proportion of sample (%)
White British	7352	93.0
Indian	94	1.2
White and Black Caribbean	82	1.0
Other White	71	0.9
White and Asian	60	0.8
Other mixed	51	0.6
Pakistani	40	0.5
White Irish	35	0.4
Caribbean	34	0.4
African	26	0.3
Other Asian	22	0.3
Other	24	0.3
Chinese	13	0.2

*Note*: Percentages refer to the proportion of participants who reported belonging to each ethnic group.

Twin‐reported demographic characteristics in adulthood (seen in Table 3 alongside CoTEDS demographics, discussed below) have changed between the ages 21 and 26 waves. The proportion of TEDS twins reporting having completed an undergraduate degree or higher has increased between ages 21 and 26 (48.7%–66.4%). The current proportion of the TEDS sample holding a university degree or higher is above the national estimates of 56.0% (Education and training statistics for the UK, 2021). The proportion of twins currently in employment (full time, part time, or self‐employed) has also increased since age 21 (52.7%–88.4%). This compares to the UK national employment rate of 84.0% for people aged 25–34 (A05 SA: Employment, 2022). A corresponding increase in monthly salaries between the two waves is also observed. The median *net* monthly salary of the TEDS twins was reported to be £1500–2000 at age 26, in line with national estimates of a median *gross* salary of £2168.45 for people aged 22–29 (Earnings and Hours, 2021). This general increase in educational attainment, employment and income is likely due to twins moving from full time education into employment.

**TABLE 3:** Representativeness of the TEDS sample in adulthood.

	Age 21 (%)	National data Age 21 (%)	Age 26 (%)	National data Age 26 (%)	CoTEDS sample at Wave 1 (%)
Highest level of qualifications					
None	0.4	–	0.3	–	2.9
1–5+ GCSEs	8.6	18.0^1^	8.0	14.0^1^	37.8
A levels	30.1	40.0^1^	14.5	20.0^1^	28.4
Undergraduate degree or higher	48.7	29.0^1^	66.5	56.0^1^	26.5
Other qualification	12.2	–	10.7	–	–
Employment					
Employed	52.7	63.4^2^	88.4	84.0^2^	61.9
Student/Trainee	32.6	–	5.6	–	4.1
Unemployed or unpaid work	6.7	7.4^2^	3.3	3.9^2^	2.4
Looking after home/family	1.1	–	1.4	–	23.6
Unable to work because of sickness of disability	–	–	1.3	–	0.9
Monthly income (median)					
	£1000–£2000	£15,00^3^	£1500–2000	£2168.25^3^	£500–1000

*Note*: Monthly income is reported for twins currently in employment. Comparative national data is reported where available. Data not available is indicated by “–“. Demographic information for the CoTEDS sample is collected at Wave 1. Mean age of twins at Wave 1 is 24.62 years (range 19.33–28.61 years). GCSEs refer to UK school qualifications in specific subjects usually taken at age 15–16 years and represent the end of compulsory schooling. A‐levels are advanced level subject specific qualifications taken between 16 and 18 years in the UK.

^1^ https://explore‐education‐statistics.service.gov.uk/data‐tables/fast‐track/1be5cf48‐fb0f‐4384‐9b55‐2e99917df8ff

^2^ https://www.ons.gov.uk/employmentandlabourmarket/peopleinwork/employmentandemployeetypes/datasets/employmentunemploymentandeconomicinactivitybyagegroupseasonallyadjusteda05sa

^3^ https://www.ons.gov.uk/employmentandlabourmarket/peopleinwork/earningsandworkinghours/datasets/agegroupashetable6

### TEDS26 – Mental health assessment

Common mental health disorders, notably anxiety and depression, pose a substantial burden for individuals, families, and society. Mental health symptoms often appear during adolescence and early adulthood (Solmi et al., 2022). What is more, rates of anxiety and comorbid anxiety‐depression are rising, especially among young people aged 18–35 years and in women (Slee et al., 2021). Consequently, research examining who is at risk for developing a mental health disorder, and how to best intervene, is essential. For these reasons, the most recent cohort‐wide wave of data collection, TEDS26, focused on mental health and wellbeing in the mid‐twenties. Combined with the wealth of developmental data, TEDS provides a unique opportunity to better understand how genetic and environmental factors influence the development and onset of mental health symptoms.

Data collection took place between July 2021 and December 2022, during which time all contactable TEDS twins were invited to take part in an online mental health assessment. Participants were offered a voucher for taking part and entered into regular prize draws. Text, email, phone, and postal reminders were sent throughout the study. The assessment was adapted from the Genetic Links to Anxiety and Depression (GLAD) Study sign up questionnaire (Davies et al., 2019). To facilitate longitudinal research and cross‐cohort collaboration, other measures were selected to mirror those used both in earlier waves of TEDS (see Table 4) and other large cohort studies. These include the Avon Longitudinal Study of Parents and Children (ALSPAC; Boyd et al., 2013), the UK Biobank Mental Health Assessment (UKBB; Davis et al., 2020), the COVID‐19 Psychiatry and Neurological Genetics study (COPING; Young et al., 2022), and the Dunedin Study (Poulton et al., 2015). A full list of measures and cohort harmonisation can be seen in Table 5.

**TABLE 4:** Mental health phenotypes assessed longitudinally in TEDS.

Age:	2	3	4	7	8	9	12	14	16	21	26
Anxiety											
Depression											
OCD symptoms											
Emotional symptoms											
Conduct problems											
Peer problems											
ADHD and hyperactivity											
Autistic traits											
Psychopathy and aggression											
Self‐harm											
Psychotic experiences											
Eating disorder symptoms											

*Note*: Measures used to assess mental health phenotypes have varied across waves depending on participant age, length of questionnaire, and overlap with other studies. Full information regarding measures can be found in the TEDS data dictionary (TEDS data dictionary, 2023).

**TABLE 5:** TEDS26 Mental Health Assessment questionnaire measures.

Assessment	Source	Reference	Overlap
Lifetime mental health			
Depression	Adapted Composite International Diagnostic Interview – Short form (CIDI‐SF)	Kessler et al. (1998)	GLAD, COPING, UKBB & AGDS
Generalised anxiety			
Specific phobia			
Social phobia			
Panic disorder			
Agoraphobia			
Mania/Hypomania	Mood Disorder Questionnaire (MDQ)	Hirschfeld et al. (2000)	GLAD
Eating disorders	Bespoke measure devised by UK Biobank team		UKBB & COPING
Personality disorders symptoms	Standardised Assessment of Severity of Personality Disorders (SASPD)	Olajide et al. (2018)	GLAD
Mental health history – Single‐item m	GLAD study	Davies et al. (2019)	GLAD & COPING
Current mental health symptoms			
Depression symptoms	Mood and Feelings Questionnaire (MFQ‐13)	Angold et al. (1995); Kroenke et al. (2001)	TEDS & ALSPAC GLAD
	Patient Health Questionnaire (PHQ‐9)		
Generalised anxiety symptoms	Severity measure of generalised anxiety disorder	Craske et al. (2013); Spitzer et al. (2006)	TEDS21GLAD & ALSPAC
	Generalised anxiety disorder assessment (GAD‐7)		
Suicidal/non‐suicidal self‐harm	Suicidal feelings	Madge et al. (2008); Paykel et al. (1974)	TEDS16 & TEDS21
	Deliberate self‐harm		
Somatic and physical health symptoms	Patient Health Questionnaire (PHQ‐15)	Kroenke et al. (2002)	GLAD
Work and social adjustment	Work and Social Adjustment Scale (WSAS)	Marks (1986)	GLAD
Emotion and behaviour	Strengths and Difficulties Questionnaire (SDQ)	Goodman (1997)	TEDS & ALSPAC
PTSD symptoms	PTSD checklist for DSM‐5 (PCL‐5)	Weathers et al. (1993)	GLAD & ALSPAC
Body dysmorphic disorder	Dysmorphic Concern Questionnaire (DCQ)	Oosthuizen et al. (1998)	GLAD (optional)
Psychotic experiences	Specific Psychotic Experiences Questionnaire (SPEQ)	Ronald et al. (2014)	TEDS16 & TEDS21
Callous‐unemotional traits	Inventory of Callous‐Unemotional Traits (ICU)	Frick (2004)	TEDS16
Hedonia subscale	Specific Psychotic Experiences Questionnaire (SPEQ)	Ronald et al. (2014)	TEDS16
Subjective wellbeing	Items selected for GLAD study		GLAD
Neurodevelopmental conditions			
Adult ADHD symptoms	Conners 3^rd^ Edition (Conners 3)	Conners (2008)	TEDS
Autistic traits ‐ adult	Ritvo Autism & Asperger Diagnostic Scale (RAADS‐14)	Eriksson et al. (2013)	GLAD & COPING
Physical health			
Physical illness history – Single item	Items from GLAD study	Davies et al. (2019)	GLAD
Chronotype/Sleep quality	Munich Chronotype Questionnaire (MCTQ)	Buysse et al. (1989); Roenneberg et al. (2003)	DunedinTEDS
	Pittsburgh Sleep Quality Index (PSQI)		
Alcohol use (lifetime and current)	Alcohol use disorders identification test (AUDIT)	Reinert and Allen (2007)	TEDS21, ALSPAC
Smoking/vaping (lifetime and current)	International Tobacco control policy Evaluation project (ITC) Youth Tobacco and E‐Cigarette survey	Heatherton et al. (1991)	TEDS16, TEDS21, ALSPAC
	Fagerström test for nicotine dependence		
Cannabis use	Cannabis Abuse Screening Test (CAST)	Cuenca‐Royo et al. (2012)	TEDS16, TEDS21, ALSPAC
Diet/Exercise	Devised by TEDS team		TEDS18, TEDS21
Hormonal contraceptive use	Devised by King's College London researchers		
Premenstrual symptoms	Premenstrual Symptoms Impact Survey (PMSIS)	Wallenstein et al. (2008)	
Environmental experiences			
Childhood traumatic events	Childhood Trauma Screener (CTS)	Glaesmer et al. (2013)	GLAD
Adulthood traumatic events	Developed by UK Biobank team	Frissa et al. (2016)	GLAD, UKBB
Domestic abuse		Khalifeh et al. (2015)	GLAD, UKBB
Life events	Coddington Life Events Scale	Coddington (1972)	TEDS16, TEDS21, ALSPAC

*Note*: This table details all measures included at the age 26 assessment in TEDS. The overlap column reflects measures that have also been included in other longitudinal cohort studies or earlier eaves of TEDS. Full information regarding measures can be found in the TEDS data dictionary (TEDS data dictionary, 2023).

The questionnaire included lifetime assessments of depression and anxiety related disorders, including generalised anxiety disorder, specific phobias, social phobias, panic disorder and agoraphobia. There were also measures assessing lifetime symptoms of mania/hypomania, eating disorders and personality disorder.

Current symptomatology was also measured for anxiety and depression. For continuity with previous waves of TEDS assessment, we included measures of current emotional and behavioural symptoms, psychotic experiences, and callous‐unemotional traits. Measures of current body dysmorphic disorder symptoms and post‐traumatic stress disorder were assessed in TEDS for the first time.

TEDS26 also incorporated measures of environmental stress relevant to mental health outcomes. These contained items relating to life events, and traumatic experiences in childhood and adulthood. We also included questions about the severity of work and social impairment relating to mental health difficulties. A single‐item measure of physical health diagnoses, measures of chronotype, sleep quality and hormonal contraceptive use were added alongside lifestyle questions relating to diet, exercise, and substance use.

Preliminary analyses of the TEDS26 data have highlighted both the high prevalence of self‐reported mental health conditions among young adults, and the importance of considering comorbidity. Of those who have taken part in the age 26 assessment, 38.6% report having previously received a mental health diagnosis from a mental health professional. Of that group, 67.3% of twins reported having received more than one diagnosis. In line with previous estimates, prevalence was higher among females (43.9%), than males (28.4%). As seen in Table 6, depression was the most frequently reported disorder with a prevalence of 25.7%. Anxiety related disorders were the next most reported diagnoses. A broad category of “anxiety, stress and nerves” had a prevalence of 24.6%, while 10.7% of twins reported diagnosis of Generalised Anxiety Disorder.

**TABLE 6:** Prevalence of self‐reported mental health and neurodevelopmental diagnoses in TEDS26.

	*N*	Proportion of sample (%)	Proportion of females (%)	Proportion of males (%)
		(95% CI)	(95% CI)	(95% CI)
Depression	1977	25.7 (24.8–26.7)	29.5 (28.2–30.8)	18.6 (17.2–20.2)
Anxiety, stress, nerves	1889	24.6 (23.6–25.5)	24.6 (23.6–25.6)	16.9 (15.5–18.3)
Generalised anxiety disorder	824	10.7 (10.1–11.4)	12.9 (12.0–13.9)	6.5 (5.6–7.5)
Panic attacks	779	10.1 (9.5–10.8)	12.6 (11.7–13.6)	5.4 (4.6–6.4)
Social anxiety	466	6.1 (5.6–6.6)	6.4 (5.7–7.1)	5.5 (4.7–6.4)
Post‐traumatic stress disorder	312	4.1 (3.6–4.5)	5.4 (4.8–6.0)	1.6 (1.2–2.1)
Obsessive compulsive disorder	250	3.3 (2.9–3.7)	3.7 (3.2–4.3)	2.4 (1.9–3.1)
Anorexia Nervosa	166	2.2 (1.9–2.5)	3.0 (2.6–3.5)	0.5 (0.3–0.9)
Other compulsive disorder	130	1.7 (1.4–2.0)	2.1 (1.8–2.6)	0.8 (0.5–1.3)
Other	115	1.5 (1.2–1.8)	1.6 (1.3–2.0)	1.2 (0.9–1.7)
Autism spectrum disorder	125	1.6 (1.4–1.9)	1.2 (0.9–1.6)	2.4 (1.9–3.1)
Attention hyperactivity disorder	127	1.6 (1.4–2.0)	1.5 (1.2–1.8)	2.0 (1.5–2.6)
Body dysmorphic disorder	110	1.4 (1.2–1.7)	2.0 (1.7–2.5)	0.3 (0.2–0.6)
Panic disorder	97	1.3 (1.0–1.5)	1.6 (1.3–2.0)	0.6 (0.4–1.0)
Personality disorder	101	1.3 (1.1–1.6)	1.8 (1.4–2.2)	0.5 (0.3–0.8)
Bulimia Nervosa	73	0.9 (0.8–1.2)	1.3 (1.0–1.7)	0.3 (0.1–0.6)
Mania/Hypomania	71	0.9 (0.7–1.2)	1.1 (0.8–1.4)	0.6 (0.4–1.0)
Specific phobia	67	0.9 (0.7–1.1)	1.0 (0.8–1.3)	0.6 (0.4–1.0)
Binge eating	57	0.7 (0.6–1.0)	1.0 (0.8–1.3)	0.2 (0.1–0.5)
Psychological overeating	46	0.6 (0.4–0.8)	0.8 (0.6–1.1)	0.2 (0.1–0.5)
Other psychotic disorder	40	0.5 (0.4–0.7)	0.6 (0.4–0.9)	0.3 (0.2–0.6)
Agoraphobia	30	0.4 (0.3–0.6)	0.5 (0.3–0.7)	0.3 (0.1–0.6)
Premenstrual dysphoric disorder	25	0.3(0.2–0.5)	0.3(0.2–0.5)	–

*Note*: Percentages refer to the proportion of participants who self‐reported having received a diagnosis from a health care professional on a single‐item mental health measure. These percentages represent lifetime prevalence rates.

### TEDS COVID‐19 study

In addition to TEDS26, recent data collection has also included the TEDS COVID‐19 Study, which aimed to capture the unique experiences of people in their 20s during the global Coronavirus pandemic declared in 2020. Planning for the COVID‐19 Study began shortly after the first UK ‘national lockdown’ in March 2020 and included four waves of data collection between April 2020 and March 2021 (Rimfeld et al., 2022). Twins contactable by email were invited to take part. Overall, 6545 twins completed at least one phase of study. Prize draws were provided as incentives for participation in this study.

To measure changes in lifestyle, behaviour and mental health resulting from this pandemic, COVID‐19 Study measures overlapped with those used at age 21. These included items relating to social support and relationships, thoughts and attitudes, substance use, mental health, and regular activities. The questionnaire also included selected items from The CoRonavIRus Health Impact Survey (CRISIS; Nikolaidis et al., 2021). A full list of measures can be found in Supplementary Table S1.

## GENOTYPE DATA

Genotype data is available for 10,346 individual TEDS twins. This subsample includes DNA from 3320 complete dizygotic twin pairs and 2666 monozygotic twins (only one member of each monozygotic twin pair was genotyped). We also hold genotype data for 1017 dizygotic twins from pairs where only one twin has been genotyped and 23 twins of unknown zygosity (see TEDS DNA Studies, 2023 for details on collections and data returns). DNA samples were collected over multiple phases, initially using cheek swabs and later using saliva kits. The samples were genotyped with Affymetrix GeneChip 6.0 (*N* = 3057), or Illumina HumanOmniExpressExome chip (*N* = 7289) (see Selzam et al., 2019 for details).

The genotyped data collected in TEDS have been used to create around 300 genome‐wide polygenic scores which are now accessible to researchers as part of the constantly updated TEDS database (see TEDS Data Dictionary, 2023 for details).

### TEDS parent DNA study

We have recently undertaken a Parent DNA Study, collecting DNA samples from the parents of TEDS twins for whom we hold genetic data. Parents designated as the contact parents throughout TEDS were invited to provide saliva samples by post. Co‐parents who also wished to take part provided contact details before being sent a kit. The parent DNA study expanded the TEDS database to include 1577 families with DNA samples from at least one parent, including DNA on 1099 parent pairs. Incorporating genomic data from multiple generations within the same family will allow researchers to control for parental genotypes while investigating the effects of genes and environments within a family.

## LINKED DATA

### National pupil database

Over the course of the study, TEDS has undertaken data linkage, or the process of linking study data to other sources of routine data, to enrich available information. Initially, this involved linking TEDS data from families who provided informed consent to data in the National Pupil Database (NPD; Department of Education, 2016), which details school characteristics, student attainment and attendance in English state‐maintained schools. This linkage was carried out in partnership with the Department for Education (DfE), and provided national educational outcome data at ages 7, 11, 14, 16 and 18 years, including exam results from the General Certificate of Secondary Education (GCSEs) at the end of compulsory schooling at age 16 and A‐Levels at age 18 (For more details see Rimfeld et al., 2018).

### Postcode data

More recently, geographical environmental data on pollution, greenspace and socio‐demographic statistics have been linked to the TEDS dataset using postcodes collected from TEDS families at the first contact, TEDS16, and TEDS21 waves of assessment (See Maxwell et al., 2022 for more details). Most of the postcodes correspond to parent addresses, apart from a small proportion of those collected at TEDS21 belonging to twins. Linked pollution and greenspace data was assessed using background pollution and land cover maps accessed from the Department of Environment Food and Rural Affairs (DEFRA; https://uk‐air.defra.gov.uk/data/pcm‐data) and UK Centre for Ecology and Hydrology (UKCEH; https://www.ceh.ac.uk/data/ukceh‐land‐cover‐maps) respectively. UK census data from the closest corresponding years were downloaded from the UK Data Service (https://ukdataservice.ac.uk/) and used to calculate socio‐demographic information of postcode areas. This included population density and measures relating to regional deprivation and socio‐economic factors.

### Linkage to participants' electronic health records

TEDS is now establishing the required conditions to extend our linked data to include National Health Service (NHS) electronic health records. Our protocols are based on the established precedents of ALSPAC (Boyd et al., 2013; Fraser et al., 2013) and TwinsUK (Moayyeri et al., 2013), and involve the NHS South London and Maudsley Clinical Data Linkage Service. Importantly, we undertook several engagement workshops with participants to co‐design the purpose and methods to link these data. TEDS has obtained the necessary permissions to trace study participants via linkage to the NHS patient register, and to provide information regarding the proposed linkage that meets UK GDPR lawfulness, fairness, and transparency principles (see Principle (a), n.d.); this information describes the intended use of participant data and enables individuals to opt‐out of this activity. Once we have traced participants, sent out study information and provided sufficient time for participant opt‐out, we will proceed to link TEDS participants to medical record databases, ranging from primary care (GP) to hospital records (e.g. A&E) and mental health services (e.g. psychological therapies service). Working with NHS data sharing agencies, the extracted NHS records will be held, processed, and analysed within an accredited and independently audited ‘Trusted Research Environment(s)’. Within these environments, this wealth of new information will be pseudonymised and combined with our existing dataset, improving our ability to ask questions about the origins and trajectories of common mental health disorders, access to and outcomes of treatment. Extractions and opt out statuses will be refreshed annually. These medical health records are being sought for all twins who were initially enrolled in the study and who have not since withdrawn (N~24,000 twins) which will allow for novel investigations of attrition in the sample and to inform statistical methods to address resulting biases.

### The UK Longitudinal Linkage Collaboration (UK LLC)

The UK LLC (https://ukllc.ac.uk) is a pan‐UK and interdisciplinary Trusted Research Environment which, through collaboration with longitudinal population studies, provides record linkage services and a ‘Trusted Research Environment’. Over 20 UK longitudinal studies are currently contributing to UK LLC ‐ where de‐personalised data relating to >280,000 participants have been systematically linked to comprehensive NHS electronic health records and records of the natural and built environment (e.g., greenspace and greenness provision, neighbourhood, and property characteristics). TEDS is actively contributing to the design of the UK LLC and is exploring options for UK LLC to provide long‐term data linkage and researcher analysis capabilities (no TEDS data are currently held in UK LLC). This will provide a means for TEDS linkages that are compatible with NHS and wider data owner requirements (i.e., an independently audited and accredited secure research environment) and to provide novel scientific opportunities resulting from integrating TEDS data into a highly heterogeneous pooled sample, including many studies of comparable ages to those in the TEDS study.

## CoTEDS

In March 2016, the TEDS team launched The Children of TEDS (CoTEDS), a project recruiting and collecting data on the children of TEDS twins. CoTEDS currently has three concurrently running waves of data collection, for parents to complete when their children are ages 1‐, 2‐, and 3 years. CoTEDS data collection corresponds with previous TEDS waves and assesses many of the same phenotypes, to enable information on parents and children to be compared across generations. This makes CoTEDS an invaluable resource to explore causal questions about intergenerational transmission of behaviour and emotion. All TEDS twins with children are eligible to join CoTEDS and are recruited during regular TEDS data collections and through social media outreach (see Ahmadzadeh et al., 2019 for details). Non‐TEDS co‐parents are also invited to take part and complete the relevant questionnaires at each wave.

The CoTEDS sample currently comprises 863 families with one TEDS twin parent and at least one child. This includes 380 trios (TEDS parent–coparent–child) and 119 families where both members of a twin pair have at least one child in CoTEDS. As seen in Table 7, children enrolled in CoTEDS range from 0 to 10+ years old. Parents who register children to CoTEDS who are older than 3 years are sent a revised version of the Wave 1 questionnaire, collecting baseline information on the pregnancy, birth, and child's health.

**TABLE 7:** Number of children registered with CoTEDS.

Child age (years)	Number
0–1	126
1–2	191
2–3	148
3–4	149
4–5	146
5–6	141
6–7	114
7–8	80
8–9	55
9–10	30
10 +	33
**Total**	**1213**

*Note*: The bold value was used to highlight the total number of children registered with the CoTEDS study.

The majority of TEDS twins with children enrolled in CoTEDS are mothers (79%). The reported ethnicity of the children in CoTEDS is 93.3% White British ethnicity. A full description of socioeconomic demographics of the CoTEDS sample can be seen in Table 3.

CoTEDS is focussed on child development, mental health, and intergenerational influences. The proportion of parents in the current CoTEDS sample who report having received a mental health diagnosis from a mental health professional are shown in Table 8. Comparison of descriptive frequencies indicate that rates of common mental health disorders, including depression and anxiety, are higher among TEDS twins enroled in CoTEDS compared to the full TEDS sample.

**TABLE 8:** Prevalence of self‐reported mental health diagnoses in TEDS twins enroled in CoTEDS.

	N	Proportion of sample (%)
		(95% CI)
Depression	285	36.3% (33.0–39.7)
Anxiety, stress, nerves & generalised anxiety disorder	246	31.3 (28.2–34.7)
Panic attacks	70	8.9 (7.1–11.1)
Social anxiety	59	7.5 (5.9–9.6)
Mania	29	3.7 (2.6–5.3)
Personality disorder	25	3.2 (2.2–4.7)
OCD	24	3.1 (2.1–4.5)

*Note*: Percentages refer to the proportion of participant who reported having received a diagnosis from a health care professional on a single‐item mental health measure.

The CoTEDS sample is growing as more TEDS twins have children. The latest wave, Wave 3, launched in February 2022 includes many repeated measures from previous waves with the addition of scales relating to gender identity and children's eating behaviours. See Supplementary Table S2 for CoTEDS measures at each wave. Future plans for the study include a Wave 4 questionnaire for school‐age children, and DNA collection from CoTEDS children and their non‐TEDS co‐parents.

## NATIONAL AND INTERNATIONAL COLLABORATIONS

In addition to core waves of assessment described above, collaborative sub‐sample studies have been undertaken in TEDS. These projects are often funded and led by external researchers but administered by the core TEDS team. More details about sub‐studies are available on the TEDS data dictionary (TEDS spin offs, 2023).

To support genome‐wide meta‐analytic work, TEDS has joined 16 genetic consortia over the past decade. These working groups cover a range of outcomes and developmental stages, including the EArly Genetics and Lifecourse Epidemiology (EAGLE; https://www.eagle‐consortium.org/) consortium, GenLang consortium (http://genlang.org/), and Major Depressive Disorder Psychiatric Genomics Consortium (MDD PGC; https://www.med.unc.edu/pgc/pgc‐workgroups/major‐depressive‐disorder/).

To further support collaboration, TEDS is included in The Catalogue for Mental Health, the largest online catalogue of mental health assessment in longitudinal studies. The catalogue provides an avenue for accessing information about the measurement of mental health and how to access data from various studies (For more information on the catalogue: https://www.cataloguementalhealth.ac.uk/).

## ACCESSING TEDS DATA

All behavioural and environmental data resulting from nearly 30 years of data collection are catalogued in the TEDS data dictionary (TEDS Data Dictionary, 2023). The data dictionary provides a detailed overview of each wave of data collection, including the sample, measures, and available scales.

TEDS data are currently free to access for researchers and students at academic research institutions. Requests must be approved by the TEDS steering committee and have the support of a core TEDS collaborator. All researchers wishing to use TEDS data are required to pre‐register analysis plans with the Open Science Framework (OSF). More information on TEDS data policy, data sharing process, and data request form can be found on our website (TEDS Data Access Policy, 2023).

After more than 25 years, TEDS remains the largest developmental twin cohort in the UK. So far, TEDS has shed light on the genetic and environmental contributions to cognition, behaviour, and psychopathology from infancy to early adulthood. However, there is still work to be done in understanding all areas of development. There are still many unknowns about the development and intergenerational transmission of mental health disorders, especially during this critical period of the mid‐twenties. We actively encourage collaborations with researchers, clinicians, and students to utilise the wealth of TEDS data to answer these questions.

## CONFLICT OF INTEREST

No authors have declared any competing or potential conflicts of interest.

## ETHICAL CONSIDERATIONS

TEDS has ethical approval from Kings College London Research Ethics Committee (References: PNM/09/10–104 and HR/DP‐20/21–22060). Consent was obtained before data collection at every wave.

## AUTHOR CONTRIBUTION

**Celestine Lockhart**: Conceptualization; Data curation; Formal analysis; Software; Visualization; Writing – original draft; Writing – review & editing. **Joanna Bright**: Data curation; Formal analysis; Project administration; Software; Writing – original draft; Writing – review & editing. **Gerome Breen**: Conceptualization; Funding acquisition; Writing – review & editing. **Andy Boyd**: Conceptualization; Funding acquisition; Writing – review & editing. **Matthew Hotopf**: Conceptualization; Funding acquisition; Writing – review & editing. **Kaili Rimfeld**: Conceptualization; Methodology; Writing – review & editing. **Margherita Malanchini**: Conceptualization; Methodology; Writing – review & editing. **Tom A. McAdams**: Conceptualization; Funding acquisition; Methodology; Project administration; Supervision; Writing – review & editing. **Robert Plomin**: Conceptualization; Funding acquisition; Methodology; Supervision; Writing – review & editing. **Thalia C. Eley**: Conceptualization; Funding acquisition; Investigation; Methodology; Project administration; Supervision; Writing – review & editing.

## Supporting information

Supporting Information S1

## Data Availability

The data used and described in this paper came from the Twins Early Development Study (TEDS). Researchers can request access to TEDS data: http://www.teds.ac.uk/researchers/teds‐data‐access‐policy.14of16‐RIMFELDETAL, however not all data from the TEDS26 Mental Health Assessment are available for use yet.
