# Supplementary material for: Twins Early Development Study (TEDS): A genetically sensitive investigation of mental health outcomes in the mid‐twenties
Source: JCPP Adv. 2023 Mar 30;3(2):e12154. doi: 10.1002/jcv2.12154 (PMC10519737; doi:10.1002/jcv2.12154)
Supplement: Supplementary file 1 — Supporting Information S1 [file JCV2-3-e12154-s001.docx]

**Supporting Information**

**Twins Early Development Study (TEDS): A genetically sensitive investigation of mental health outcomes in the mid-twenties.**

Celestine Lockhart^1 (0000-0002-6584-3696)^, Yasmin Ahmadzadeh^1 (0000-0003-1333-1661)^,

Gerome Breen^1,9 (0000-0003-2053-1792)^, Joanna Bright^1 (0000-0002-0695-0491)^,

Shannon Bristow^1 (0000-0002-0896-781X)^, Andy Boyd^2 (0000-0002-8614-3728)^, Johnny Downs^3,4 (0000-0002-8061-295X)^, Matthew Hotopf^4,5 (0000-0002-3980-4466)^, Elisavet Palaiologou^1 (0000-0003-4707-3349)^,

Kaili Rimfeld^1,6 (0000-0001-5139-065X)^, Jessye Maxwell^1(0000-0002-3158-7479)^,

Margherita Malanchini^1,7 (0000-0002-7257-6119)^, Tom A. McAdams^1,8 (0000-0002-6825-3499)^,

Robert Plomin^1 (0000-0002-0756-3629)^ & Thalia C. Eley^1,9 (0000-0001-6458-0700)^

1. Social, Genetic and Developmental Psychiatry Centre, Institute of Psychiatry, Psychology and Neuroscience, King's College London, Denmark Hill, Camberwell, London, UK
2. Population Health Sciences Institute, Bristol Medical School, University of Bristol, Bristol, UK
3. Department of Child and Adolescent Psychiatry, Institute of Psychiatry, Psychology & Neuroscience, King’s College London
4. South London and Maudsley NHS Foundation Trust, London, UK
5. Department of Psychological Medicine, Institute of Psychiatry Psychology and Neuroscience, King’s College London, London, UK
6. Department of Psychology, Royal Holloway University of London, Egham, Surrey, UK
7. Queen Mary University of London, London, UK
8. Promenta Research Centre, University of Oslo, Oslo
9. UK National Institute for Health Research (NIHR) Biomedical Research Centre, South London and Maudsley Hospital, London, UK

**Correspondence to**

Prof Thalia Eley, [thalia.eley@kcl.ac.uk](mailto:thalia.eley@kcl.ac.uk), 020 7848 0863

Room C1.16, Social, Genetic and Developmental Psychiatry Centre, Institute of Psychiatry, Psychology & Neuroscience, King’s College London, London, United Kingdom

**Contents**

Tables

**Table S1**. Complete list of measures included in the TEDS COVID-19 Study.

**Table S2**. Complete list of measures included in each wave of the CoTEDS project.

Figures

**Figure S1**. Overall trends of sample sizes in TEDS across core waves of data collection.

| Table S1. TEDS Covid Study Questionnaire Measures | | |
| --- | --- | --- |
| Assessment | Source | Reference |
| Living environment – people in the home | The CoRonavIrus Health Impact Survey (CRISIS) | Nikolaidis et al., (2021) |
| Coronavirus exposure |  |  |
| Worries about health |  |  |
| Lifestyle changes (Employment, education, worries, sleep patterns) |  |  |
| Parent contact | Items created by TEDS researchers |  |
| Love and relationships | Contentment with Life Assessment Scale (CLAS) | Lavallee et al., (2007) |
| Leisure and hobbies |  |  |
| Community |  |  |
| Intimate partner violence | Center for Disease Control and Prevention Violence Prevention questionnaire | Basile et al., (2007) |
| Peer victimisation | Multidimensional Peer Victimisation Scale | Mynard & Joseph (2000) |
| Life goals | GOALS | Pöhlmann & Brunstein (1997) |
| Purpose in life |  | Crumbaugh & Maholick (1964) |
| Attitudes towards health care provision | NatCen Social Research's British Social Attitudes (BSA) Survey | <http://natcen.ac.uk/our-research/research/british-social-attitudes/> |
| Financial literacy | OECD instrument for financial literacy | <https://www.oecd.org/finance/financial-education/49319977.pdf> |
| Life events | Adapted Coddington Life Events Scale | Coddington (1972) |
| Alcohol consumption | Adapted Alcohol Use Disorders Identification Test (AUDIT) |  |
| Smoking and vaping |  |  |
| Cannabis use | Cannabis Abuse Screening Test (CAST) | Legleye et al., (2011) |
| Emotion and behaviour | Strengths and Difficulties Questionnaire (SDQ) | R. Goodman (1997) |
| General anxiety | Severity Measure for Generalised Anxiety Disorder | Craske et al., (2013) |
| Depression symptoms | Short Mood and Feelings Questionnaire (SMFQ) | Angold et al., (1995) |
| Self-harm | Suicidal Feelings | Paykel et al., (1974) |
| Physical activity | Devised by TEDS team |  |
| Media and technology use | Media and Technology Usage and Attitudes Scale | Rosen et al., (2013) |
| Volunteering | Questions taken from the ALSPAC study age 20 booklet |  |

| Table S2. CoTEDS Questionnaire Measures |  |  |  |
| --- | --- | --- | --- |
| Assessment | Wave | Overlap | Reference |
| Child Eating Behaviours Questionnaire | 3 |  | Wardle et al., (2001) |
| CoTEDS breastfeeding battery | 1,2 | TEDS, EGDS, ALSPAC |  |
| Brief Infant Sleep Questionnaire | 1,2 |  | Sadeh (2004) |
| CoTEDS sleep problem scale | 3 | TEDS | [TEDS](https://www.teds.ac.uk/datadictionary/pdfs/3yr/3yr_parent_booklet.pdf) |
| Denver Developmental Screening | 1,2 | ALSPAC | Frankenburg & Dodds (1967) |
| Parent Report of Children’s Abilities (PARCA) - parent assessed at wave 2 only | 2,3 |  | Saudino et al., (1998) |
| CoTEDS language items | 2,3 | TEDS | Fenson et al., (1994) |
| Parent Report Gender Identity Questionnaire | 3 |  | Johnson et al., (2004) |
| Emotionality, Activity and Shyness | 1,2,3 | MoBa | Buss & Plomin (1984) |
| Infant Toddler Social and Emotional Assessment | 1,2,3 | MoBa | Briggs-Gowan & Carter (1998) |
| Infant Characteristics Questionnaire | 1,2 | EGDS, MoBa | Bates et al., (1979) |
| Strengths and Difficulties Questionnaire | 2,3 | TEDS, ALSPAC | A. Goodman & Goodman (2009) |
| Confusion Hubbub and Order Scale | 1,3 | TEDS | Matheny Jr et al., (1995) |
| CoTEDS parent sleep items & shift work | 1,2 |  |  |
| Pittsburg Sleep Quality Index | 1,2 | TEDS, EGDS | Buysse et al., (1989) |
| Family Finance Questionnaire & other socioeconomic items | 1,3 | TEDS, EGDS | [ONS](https://www.ons.gov.uk/), Conger et al., (1994) |
| CoTEDS 'support from others' items | 1,2 |  |  |
| World Health Organization’s Quality of Life instrument | 1,2 | MoBa | The WHOQOL Group (1998) |
| Behaviour Affect Rating Scale | 1,2,3 | EGDS | Cui et al., (2005) |
| CoTEDS substance use items | 2 |  | Tracy et al., (2017) |
| Alcohol Use Disorders Identification Test | 2 | TEDS, MoBa | Saunders et al., (1993) |
| History Major Depression | 1 | MoBa | Kendler et al., (1993) |
| Short Mood and Feelings Questionnaire | 2,3 | ALSPAC | Angold et al., (1995) |
| Revised Child Anxiety and Depression Scale (adult version) | 2,3 | G1219 | Chorpita et al., (2005) |
| Edinburgh Postnatal Depression | 1 | TEDS, EGDS | Cox et al., (1987) |
| Generalised Anxiety Disorder-7 | 2,3 | UKBB | Spitzer et al., (2006) |
| Affective Reactivity Index | 2,3 |  | Stringaris et al., (2012) |
| Therapy service use | 1,2,3 | EGDS |  |
| CoTEDS play & screen time items | 1,2,3 |  | Ahmadzadeh et al., (2019) |
| Baby Care Questionnaire | 1 |  | Winstanley & Gattis (2013) |
| Parental Cognitions & Conduct Towards Infant Scale (PACOTIS) | 1,2,3 |  | Boivin et al., (2005) |
| Parental Feelings Questionnaire | 1,2,3 | TEDS | Deater-Deckard (2000) |
| Parent Infant Caregiving Touch | 1,2 |  | Koukounari et al., (2015) |
| Parenting Daily Hassles | 1,2,3 | EGDS | Crnic & Greenberg (1990) |
| The Parenting Scale | 2,3 |  | Arnold et al., (1993) |
| Pregnancy & birth | 1 |  |  |
| CoTEDS health items | 1,2,3 |  |  |

**Figure S1**. Overall trends of sample sizes in TEDS across core waves of data collection.


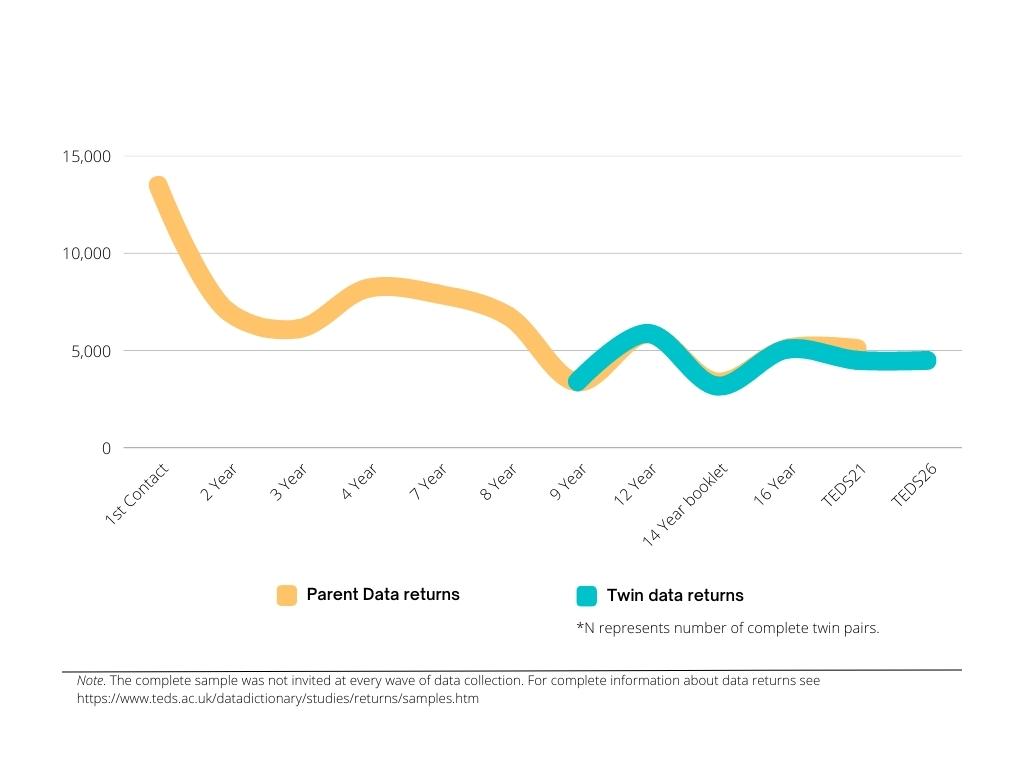


**References**

Ahmadzadeh, Y., Lester, K., Oliver, B., & Mcadams, T. (2019). *The Parent Play Questionnaire (PPQ): Development of a parent questionnaire to assess parent-child play and digital media use*. https://doi.org/10.31234/osf.io/5yqu7

Angold, A., Costello, E. J., Messer, S. C., & Pickles, A. (1995). Development of a short questionnaire for use in epidemiological studies of depression in children and adolescents. *International Journal of Methods in Psychiatric Research*, *5*, 237–249.

Arnold, D. S., O’Leary, S. G., Wolff, L. S., & Acker, M. M. (1993). The Parenting Scale: A measure of dysfunctional parenting in discipline situations. *Psychological Assessment*, *5*, 137–144. https://doi.org/10.1037/1040-3590.5.2.137

Basile, K. C., Hertz, M. F., & Back, S. E. (2007). *Intimate Partner Violence and Sexual Violence Victimization Assessment Instruments for Use in Healthcare Settings: (410572008-001)* [Data set]. American Psychological Association. https://doi.org/10.1037/e410572008-001

Bates, J. E., Freeland, C. A. B., & Lounsbury, M. L. (1979). Measurement of Infant Difficultness. *Child Development*, *50*(3), 794–803. https://doi.org/10.2307/1128946

Boivin, M., Pérusse, D., Dionne, G., Saysset, V., Zoccolillo, M., Tarabulsy, G. M., Tremblay, N., & Tremblay, R. E. (2005). The genetic-environmental etiology of parents’ perceptions and self-assessed behaviours toward their 5-month-old infants in a large twin and singleton sample. *Journal of Child Psychology and Psychiatry, and Allied Disciplines*, *46*(6), 612–630. https://doi.org/10.1111/j.1469-7610.2004.00375.x

Briggs-Gowan, M. J., & Carter, A. S. (1998). Preliminary acceptability and psychometrics of the infant–toddler social and emotional assessment (ITSEA): A new adult-report questionnaire. *Infant Mental Health Journal: Official Publication of The World Association for Infant Mental Health*, *19*(4), 422–445.

Buss, A. H., & Plomin, R. (1984). *Early Developing Personality Traits*. 196.

Buysse, D. J., Reynolds, C. F., Monk, T. H., Berman, S. R., & Kupfer, D. J. (1989). The Pittsburgh sleep quality index: A new instrument for psychiatric practice and research. *Psychiatry Research*, *28*(2), 193–213. https://doi.org/10.1016/0165-1781(89)90047-4

Chorpita, B. F., Moffitt, C. E., & Gray, J. (2005). Psychometric properties of the Revised Child Anxiety and Depression Scale in a clinical sample. *Behaviour Research and Therapy*, *43*(3), 309–322. https://doi.org/10.1016/j.brat.2004.02.004

Coddington, R. D. (1972). The significance of life events as etiologic factors in the diseases of children—II a study of a normal population. *Journal of Psychosomatic Research*, *16*(3), 205–213.

Conger, R. D., Ge, X., Elder, G. H., Lorenz, F. O., & Simons, R. L. (1994). Economic Stress, Coercive Family Process, and Developmental Problems of Adolescents. *Child Development*, *65*(2), 541–561. https://doi.org/10.2307/1131401

Cox, J. L., Holden, J. M., & Sagovsky, R. (1987). Detection of postnatal depression. Development of the 10-item Edinburgh Postnatal Depression Scale. *The British Journal of Psychiatry: The Journal of Mental Science*, *150*, 782–786. https://doi.org/10.1192/bjp.150.6.782

Craske, M., Wittchen, U., Bogels, S., Stein, M., Andrews, G., & Lebeu, R. (2013). Severity measure for generalized anxiety disorder-adult. *American Psychiatric Association*.

Crnic, K. A., & Greenberg, M. T. (1990). Minor Parenting Stresses with Young Children. *Child Development*, *61*(5), 1628–1637. https://doi.org/10.2307/1130770

Crumbaugh, J. C., & Maholick, L. T. (1964). An experimental study in existentialism: The psychometric approach to Frankl’s concept of noogenic neurosis. *Journal of Clinical Psychology*, *20*(2), 200–207.

Cui, M., Lorenz, F. O., Conger, R. D., Melby, J. N., & Bryant, C. M. (2005). Observer, Self-, and Partner Reports of Hostile Behaviors in Romantic Relationships. *Journal of Marriage and Family*, *67*(5), 1169–1181. https://doi.org/10.1111/j.1741-3737.2005.00208.x

Deater-Deckard, K. (2000). Parenting and Child Behavioral Adjustment in Early Childhood: A Quantitative Genetic Approach to Studying Family Processes. *Child Development*, *71*(2), 468–484. https://doi.org/10.1111/1467-8624.00158

Development of the World Health Organization WHOQOL-BREF quality of life assessment. The WHOQOL Group. (1998). *Psychological Medicine*, *28*(3), 551–558. https://doi.org/10.1017/s0033291798006667

Fenson, L., Dale, P. S., Reznick, J. S., Bates, E., Thal, D. J., & Pethick, S. J. (1994). Variability in early communicative development. *Monographs of the Society for Research in Child Development*, *59*(5), 1–173; discussion 174-185.

Frankenburg, W. K., & Dodds, J. B. (1967). The Denver Developmental Screening Test. *The Journal of Pediatrics*, *71*(2), 181–191. https://doi.org/10.1016/S0022-3476(67)80070-2

Goodman, A., & Goodman, R. (2009). Strengths and difficulties questionnaire as a dimensional measure of child mental health. *Journal of the American Academy of Child and Adolescent Psychiatry*, *48*(4), 400–403. https://doi.org/10.1097/CHI.0b013e3181985068

Goodman, R. (1997). The Strengths and Difficulties Questionnaire: A research note. *Journal of Child Psychology and Psychiatry*, *38*(5), 581–586.

Johnson, L. L., Bradley, S. J., Birkenfeld-Adams, A. S., Kuksis, M. A. R., Maing, D. M., Mitchell, J. N., & Zucker, K. J. (2004). A parent-report gender identity questionnaire for children. *Archives of Sexual Behavior*, *33*(2), 105–116.

Kendler, K. S., Neale, M. C., Kessler, R. C., Heath, A. C., & Eaves, L. J. (1993). The Lifetime History of Major Depression in Women: Reliability of Diagnosis and Heritability. *Archives of General Psychiatry*, *50*(11), 863–870. https://doi.org/10.1001/archpsyc.1993.01820230054003

Koukounari, A., Pickles, A., Hill, J., & Sharp, H. (2015). Psychometric Properties of the Parent-Infant Caregiving Touch Scale. *Frontiers in Psychology*, *6*. https://doi.org/10.3389/fpsyg.2015.01887

Lavallee, L. F., Hatch, P. M., Michalos, A. C., & McKinley, T. (2007). Development of the contentment with life assessment scale (CLAS): Using daily life experiences to verify levels of self-reported life satisfaction. *Social Indicators Research*, *83*(2), 201–244.

Legleye, S., Janssen, E., Beck, F., Chau, N., & Khlat, M. (2011). Social gradient in initiation and transition to daily use of tobacco and cannabis during adolescence: A retrospective cohort study. *Addiction*, *106*(8), 1520–1531.

Matheny Jr, A. P., Wachs, T. D., Ludwig, J. L., & Phillips, K. (1995). Bringing order out of chaos: Psychometric characteristics of the confusion, hubbub, and order scale. *Journal of Applied Developmental Psychology*, *16*(3), 429–444.

Mynard, H., & Joseph, S. (2000). Development of the multidimensional peer-victimization scale. *Aggressive Behavior: Official Journal of the International Society for Research on Aggression*, *26*(2), 169–178.

Nikolaidis, A., Paksarian, D., Alexander, L., Derosa, J., Dunn, J., Nielson, D. M., Droney, I., Kang, M., Douka, I., Bromet, E., Milham, M., Stringaris, A., & Merikangas, K. R. (2021). The Coronavirus Health and Impact Survey (CRISIS) reveals reproducible correlates of pandemic-related mood states across the Atlantic. *Scientific Reports*, *11*(1), 1. https://doi.org/10.1038/s41598-021-87270-3

Paykel, E. S., Myers, J. K., Lindenthal, J. J., & Tanner, J. (1974). Suicidal feelings in the general population: A prevalence study. *The British Journal of Psychiatry*, *124*(582), 460–469.

Pöhlmann, K., & Brunstein, J. C. (1997). GOALS: Ein Fragebogen zur Messung von Lebenszielen. *Diagnostica*.

Rosen, L. D., Whaling, K., Carrier, L. M., Cheever, N. A., & Rokkum, J. (2013). The Media and Technology Usage and Attitudes Scale: An empirical investigation. *Computers in Human Behavior*, *29*(6), 2501–2511. https://doi.org/10.1016/j.chb.2013.06.006

Sadeh, A. (2004). A brief screening questionnaire for infant sleep problems: Validation and findings for an Internet sample. *Pediatrics*, *113*(6), e570-577. https://doi.org/10.1542/peds.113.6.e570

Saudino, K. J., Dale, P. S., Oliver, B., Petrill, S. A., Richardson, V., Rutter, M., Simonoff, E., Stevenson, J., & Plomin, R. (1998). The validity of parent-based assessment of the cognitive abilities of 2-year-olds. *British Journal of Developmental Psychology*, *16*, 349–363. https://doi.org/10.1111/j.2044-835X.1998.tb00757.x

Saunders, J. B., Aasland, O. G., Babor, T. F., de la Fuente, J. R., & Grant, M. (1993). Development of the Alcohol Use Disorders Identification Test (AUDIT): WHO Collaborative Project on Early Detection of Persons with Harmful Alcohol Consumption--II. *Addiction (Abingdon, England)*, *88*(6), 791–804. https://doi.org/10.1111/j.1360-0443.1993.tb02093.x

Spitzer, R. L., Kroenke, K., Williams, J. B., & Löwe, B. (2006). A brief measure for assessing generalized anxiety disorder: The GAD-7. *Archives of Internal Medicine*, *166*(10), 1092–1097.

Stringaris, A., Goodman, R., Ferdinando, S., Razdan, V., Muhrer, E., Leibenluft, E., & Brotman, M. A. (2012). The Affective Reactivity Index: A concise irritability scale for clinical and research settings. *Journal of Child Psychology and Psychiatry, and Allied Disciplines*, *53*(11), 1109–1117. https://doi.org/10.1111/j.1469-7610.2012.02561.x

Tracy, D. K., Wood, D. M., & Baumeister, D. (2017). Novel psychoactive substances: Types, mechanisms of action, and effects. *BMJ (Clinical Research Ed.)*, *356*, i6848. https://doi.org/10.1136/bmj.i6848

Wardle, J., Guthrie, C. A., Sanderson, S., & Rapoport, L. (2001). Development of the Children’s Eating Behaviour Questionnaire. *Journal of Child Psychology and Psychiatry, and Allied Disciplines*, *42*(7), 963–970. https://doi.org/10.1111/1469-7610.00792

Winstanley, A., & Gattis, M. (2013). The Baby Care Questionnaire: A measure of parenting principles and practices during infancy. *Infant Behavior & Development*, *36*(4), 762–775. https://doi.org/10.1016/j.infbeh.2013.08.004
